# Replenishment of the Gas in a Hydrophobically-Structured Surface by Mass Transfer at the Liquid-Gas Interface for Improving the Stability of Entrapped Gas

**DOI:** 10.3390/mi13111893

**Published:** 2022-11-02

**Authors:** Bao Wang, Caihua Wang, Ding Weng, Marisa Lazarus, Dayun Yan, Xiaoyan Liu

**Affiliations:** 1 Department of Mechanical Engineering, Northeast Petroleum University, Daqing 163318, China; 2 State Key Laboratory of Tribology, Tsinghua University, Beijing 100084, China; 3 Department of Physics, George Washington University, Washington, DC 20052, USA; 4 Department of Mechanical and Aerospace Engineering, George Washington University, Washington, DC 20052, USA

**Keywords:** hydrophobic, flow, evaporation, micro-grooved surface, entrapped gas

## Abstract

The underwater nonwetted state on a superhydrophobic surface is hardly maintained in flowing water because the entrapped gas dissolves into the water or is carried off by flow. Therefore, a source gas is necessary to maintain a superhydrophobic state for its applications under realistic conditions. As detailed in this paper, based on the gas entrapped on a hydrophobic structured surface, the gas regeneration was experimentally achieved to replenish the losses of gas carried off by the flowing and reduced through dissolution. Furthermore, the mechanism of mass transfer at the liquid-gas interface was investigated by simulation. The results indicated that water molecules at a liquid-gas interface should escape to entrapped gas when water content didn’t reach saturation. This phenomenon could be due to the evaporation at the liquid-gas interface. With the increasing water content in the entrapped gas, the evaporation rate at the liquid-gas interface descended gradually. Under the action of flowing, the substances containing high concentrations of water molecule was washed away at the liquid-gas interface. Therefore, the low concentration of the water molecule at the liquid-gas interface was created. As a result, the equilibrium of water and gas at the liquid-gad interface was broken, and the evaporation continued to replenish the lost gas. Overall, the presented results in this study could be considered a promising candidate for replenishing the lost gas in hydrophobic structured surfaces by mass transfer at the liquid-gas interface.

## 1. Introduction

Recently, the wettability of solid surfaces, particularly superhydrophobic surfaces with a big contact angle (>150°), has attracted extensive attention. When submerged in water, these superhydrophobic surfaces can sustain gas between their surface microstructures, resulting in a layer of gas found at the solid-liquid interface. Therefore, these surfaces are understood to contain a superior alternative gas-sustaining phase that achieves an obvious slip with a length ranging from several micrometers to hundreds [1,2,3,4,5]. Experiments utilizing these superhydrophobic surfaces have achieved drag-reduction rates of 5–30% [6,7,8]. These common superhydrophobic surfaces can hold air pockets (in rough structures) and have demonstrated an effective slip for underwater drag-reduction [9]. However, it is still difficult to sustain an air-pocket stability, particularly under conditions where the liquid is flowing over the surface at a high speed or under a liquid pressure [10,11,12,13,14,15,16]. In these realistic conditions, no superhydrophobic surface has shown nonwetting properties underwater [17] because this requires first that the surface maintains a no-wetted state. Therefore, drag reduction in a superhydrophobic surface has never been achieved under realistic conditions [14]. In flowing water, higher flow velocities result in fast surface air layer removal rates [11,15]. Aljallis et al., experimentally indicated that the air was dissipated from the superhydrophobic surface in shear flow [18]. Furthermore, the air layer between surface structures becomes unstable immediately by the liquid pressure or over time by gas diffusion, leading to the collapse of the gas layer and the filling in of the surface structures with the surrounding water [13]. Theoretically, the collapse pressure at which the wetting transition occurs is inversely proportional to the pitch of surface structures [13]. In agreement with theoretical prediction, it was shown that the wetting transition occurred at varying liquid pressure ranging from 5 to 30 kPa above the ambient, depending on microstructures [13].

Once the gas layer is disrupted, the system becomes ineffective, and the gas loss at the liquid-solid interface is irreversible. Without any energy or gas-providing device, the loss of trapped gas to the surrounding liquid by diffusion is inevitable [13]. To counteract this, a lubricating gas film can be generated by vaporizing liquid at the surface at a temperature above the Leidenfrost point or by producing bubbles at the interface [19,20,21,22]. However, these methods require continuous energy input or a gas-providing device, both of which are active methods, limiting their application in practical conditions.

As passive methods, both evolution and evaporation of dissolved gas may generate gas underwater. During the evolution of dissolved gas, the time required for a gas bubble to grow ten times in size is several hundred seconds, even when the ratio of the dissolved gas concentration to the dissolved gas concentration for a saturated solution is 1.25 [23]. The growth rate of gas by evolution is too slow to achieve an effective gas layer on a solid surface underwater. Therefore, the evolution of dissolved gas has never been used to provide gas. On the other hand, evaporation may provide a feasible method to generate gas. Evaporation rate is inversely proportional to the concentrations of water molecules in the gas. Generally, concentrations of water molecules increase as the liquid vaporizes until it reaches a water-saturated state. However, suppose the concentrations of water molecules in the entrapped gas near the liquid-gas interface could be kept low. In that case, evaporation should be a relatively more violent and effective phenomenon to generate gas.

As detailed in this paper, water flow was considered essential to wash away the gas with high-concentration of the water molecule. This should achieve the sustained low concentration of water molecule needed to enable rapid evaporation at the liquid-gas interface. The mechanism of mass transfer at the liquid-gas interface was investigated by numerical simulation, and the experiments were conducted to verify the proposed approach. The state on the hydrophobic structured surface was directly observed under the flowing water condition. These results have surpassed prior limitations of the evaporation method at the liquid-gas interface for gas generation in flowing water.

## 2. Materials and Methods

### 2.1. Theoretical Evaporation Rate

Evaporation at the liquid-gas interface is a process of mass transfer. This phenomenon should be considered an important means of replenishing the gas for immersed hydrophobic surfaces [24]. Hertz conducted evaporation experiments and was the first to state an upper limit to the maximum rate of evaporation (Hertz [24], as cited by Eames et al.):

(1) $$n=\frac{1}{4}N\bar{C},$$

where n is the number of impacts per unit area per unit time ($\mathrm{kmol}\cdot m^{-2}\;s^{-1}$), *N* is the number of molecules per volume ($\mathrm{kmol}\cdot m^{-3}$), and $\bar{C}\;$ is the arithmetic mean of the velocities of the molecules.

From Maxwell’s law of velocity distribution of molecules

(2) $$\bar{C}={\left(\frac{8k_{B}T_{s}}{\pi m}\right)}^{1/2},$$

where $K_{B}$ is Boltzmann’ constant $1.38\times 10^{-23}\;{\mathrm{JK}}^{-1}$, $T_{S}\;$ is liquid surface temperature, and *m* is the mass of each molecule.

And from the Idea Gas Laws [25]:

(3) $$P_{S}V=mk_{B}T_{S},$$

where *P*_s_ is gas pressure, and *V* is gas volume. And in a unit volume of gas:

(4) $$N=\frac{P_{s}}{k_{B}T_{s}},$$

Substituting for N and $\bar{C}$ in Equation (1)

(5) $$n=\frac{P_{s}}{{\left(2\pi mk_{B}T_{s}\right)}^{1/2}},$$

When some water molecules escaped from the liquid-gas interface, others in gas entered the interface; effectively, evaporation and condensation happened simultaneously. Therefore, the evaporation rate should be modified to give Equation (6).

(6) $$n=\frac{1}{{\left(2\pi mk_{B}\right)}^{1/2}}\left(\frac{P_{s}}{\sqrt{T_{s}}}-\frac{P_{v}}{\sqrt{T_{v}}}\right),$$

The evaporation coefficient of water has been the subject of many theoretical and experimental papers and is still an area of dispute. Part of the reason for this intense interest is that some liquids, water included, appear to evaporate at a rate far less than the theoretical rate calculated using Knudsen’s equation. However, this approach can be used to analyze the factors contributing to evaporation at the liquid-gas interface.

### 2.2. Preparation of the Hydrophobic Structured Surface

To obtain entrapped gas in structures for enough time to provide the necessary premise for the evaporation at liquid-gas interface, the gas must not be completely carried away by flowing water [26]. Therefore, a hydrophobic transverse microgrooved (HTM) structure was designed to achieve our goal of sustaining an amount of gas in gaps [27]. Furthermore, when water perpendicularly flows over our designed HTM surface, gases are blocked by ridges and cannot escape easily.

**Figure 1:**
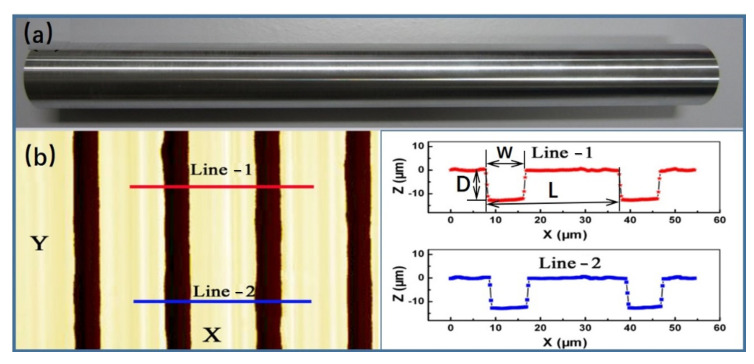
(color online) (**a**) Sample with HTMs with an outer diameter of 39 mm and length of 325 mm, (**b**) surface topography measurement of microgrooved surface at the position of line-1 and line-2, the profile of the microgroove had a cross section with an isosceles trapezoidal shape with a height (depth: D) of 12 μm, groove width (W) of 10 μm, and pitch (L) between two neighboring grooves of 30 μm.

In addition to its hydrophobicity, the physical surface characteristics of our design contributed to sustaining gas in the gaps. In our experiments, the turning method fabricated a series of transverse microgrooves, and the surface topographies were measured (Figure 1). The outer diameter of the tube sample was 39 mm, and the turning length was 325 mm. The profile of the microgroove had a cross-section with an isosceles trapezoidal shape with a height (depth: D) of 12 μm), groove width (W) of 10 μm, and pitch (L) between two neighboring grooves of 30 μm. The roughness of the platform between two neighboring microgrooves was the same as that of the smooth surface, which was used as a comparison (roughness was smaller than 0.8 μm).

The original smooth surface had a contact angle of 65°, which was hydrophilic, prior to treatment. To improve the water repellency of the surface, a coating of a low-surface-energy material named fluoroalkyl silane (FAS-17) [27,28] was applied. Based on contact-angle measurements of the corresponding profile of water droplets (5 μL) on the smooth samples modified by this low-surface-energy material, we ascertained that the FAS-17 coating can achieve a contact angle of 115 ± 2° on the smooth surface and an angle of about 130° on the hydrophobic micro-grooved surface.

According to the previous investigation, the intruding angle β (the angle between the tangent of a three-phase junction and the horizontal line), which was an acritical parameter, was proposed for the entrapped gas in the surface profile underwater [29]. To achieve a Cassies state, the intruding angle should be less than the maximum asperity slope angle. Based on the profile information of a water droplet on the surface shown in Table 1, the intruding angle for a droplet is described as:

(7) $$\beta =\pi -\theta_{0}+\arcsin\left\{\frac{f_{LG}}{\lambda }\frac{1}{\sigma } [\frac{2\sigma }{R_{D}}+\rho gR_{D}(1-\cos\theta )]\right\},$$

where, *θ*_0_ is the true contact angle; *f*_LG_ is the ratio of the projected area of the liquid-gas interface to the apparent contact area under the droplet; λ is the contact line density, the length of the contact line over the entrapped gas per unit apparent contact area; 2σ/R_D_ is pressure induced by the surface tension at the apex of droplet; R_D_ is the radius of the droplet; and θ is the apparent contact angle. The true contact angle can be considered as the contact angle on the original smooth surface coated with FAS-17, which is 115°. The apparent contact angle, 128°, is the contact angle on the hydrophobic grooved surface. *f*_LG_ is 33.3%; λ is 6.67 × 104 1/m; σ is 0.0727 N/m, liquid density is 1003 kg/m^3^; R_D_ is 1.2 mm. Thus, the intruding angle of, 65.5°, was less than the maximum asperity slope angle (π/2 − α). Thus, the three-phase lines of contact didn’t enter the grooves (If the calculated intruding angle exceeds the maximum, the liquid-gas interface will not keep balance, which will result in the entrance of liquid into grooves). Additionally, according to the literature [27], initial gas can be entrapped in the above microgrooves on the surface, which was proved by optical measurements [27].

### 2.3. Experimental Methods

**Figure 2:**
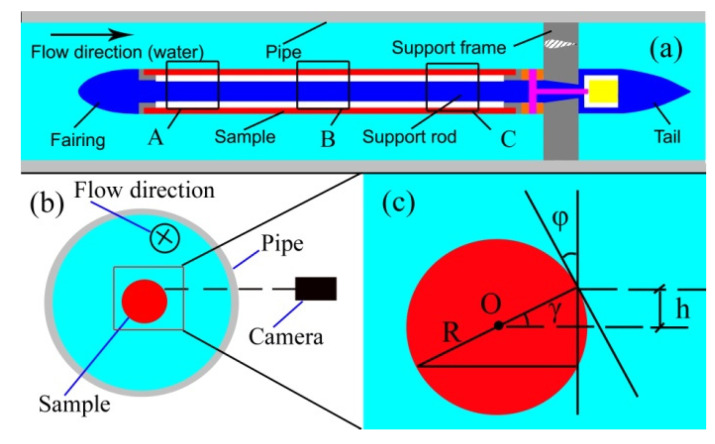
(color online) (**a**) Schematic diagrams of the water tunnel used for optical measurements of gas, A, B, and C are three positions for observation, (**b**) the position of camera, which is a sectional view of (**a**,**c**) schematic diagram of the glancing angle, R is the radius of the sample, h is the offset distance from the camera.to the center line of sample, φ is a glancing angle was equal to another angle γ.

An optical observation was performed to evaluate the effectiveness of our approach when water flowed over the HTM surface in a water tunnel with a closed circulation system. Visualizations of gas on samples were conducted in a test section of the water tunnel, as shown in Figure 2a, which was transparent (Plexiglas^®^, Beijing, China, with an inner diameter of 120 mm and thickness of 15 mm) for observation. As shown in Figure 2, three positions (A, B and C) were selected to observe. When the superhydrophobic surface (sustaining the gas between structures) was immersed in water and viewed at a glancing angle, it appeared to have high brightness [30,31,32]. According to the optical property, there was a distance (h) between the camera and the sample’s center. Therefore, a glancing angle (φ) was achieved. Based on the geometrical calculations, the glancing angle was equal to another angle γ, which indicates that the height (h) determines the glancing angle. Therefore, we can obtain an appropriate glancing angle for our observations by only moving the camera in the vertical direction.

During the measurements, water flowed over the surface in the test section. Therefore, the intensity of white light reflected from the submerged surface was measured as a description of gas. The water used in the experiment was tap water, and its temperature in the tunnel was 20 ± 2 °C.

### 2.4. Numerical Simulation

Here, a two-dimensional model was established to simulate the shape of the gas-liquid interface under dynamic conditions. The flow on the grooved surface is fast-strained, and the unsteady state-based renormalized group (RNG) k-ε model was used to improve the accuracy of numerical simulation. The implicit solution scheme combined algebraic multigrid methods to achieve faster convergence. The discretization scheme of all equations adopted the second-order upwind scheme to obtain higher result accuracy. The velocity-pressure coupling was established through the pressure-velocity correlation using the SIMPLE (semi-implicit method for pressure-linked equation) algorithm. Residuals were continuously monitored for continuity, x-velocity, y-velocity, k, and ε. The calculation time step was set to 5 × 10^−8^ s, and the total calculation time was greater than 0.5 ms. According to the geometric characteristics of the designed grooved surface, a series of regularly distributed grooves was selected as the research object, and the periodic boundary condition was set to simulate the actual situation. In this model, the designed surface and the smooth surface were located on the upper and the lower walls of the channel. The no-slip condition was adopted for the smooth surface so that the drag on the smooth surface and the hydrophobic grooved surface under the same conditions can be calculated at the same time. The height of the calculation domain was H = 450 µm, which was proven to be high enough to ensure the boundary layers of the upper and lower walls did not affect each other. Sufficient grid density was ensured in the area near the wall, and unstructured grids were used to densify the area near the groove wall to ensure calculation accuracy. The total number of grids used was 71,046. After the grid independence verification, the grid was obtained.

## 3. Results and Discussion

### Observation of Gases

**Figure 3:**
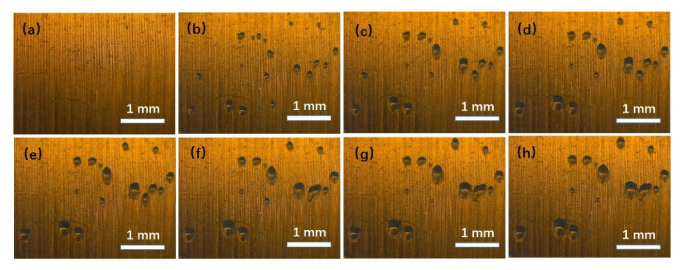
(color online) The state of gas at the submerged hydrophobic structured surface under static conditions at different time; (**a**) 0 s; (**b**) 150 s; (**c**) 300 s; (**d**) 450 s; (**e**) 600 s; (**f**) 750 s; (**g**) 900 s; (**h**) 1050 s.

When the hydrophobic structured surface was immersed in static water, the partial pressure of water (Pv) in the entrapped gas was lower than the saturated vapor pressure (Ps), because the entrapped gas was carried from the atmosphere. According to Equation (5), evaporation at the liquid-gas interface occurred. Accompanied by the evaporation, the gas ascended until the partial water pressure reached the saturated vapor pressure. As shown in Figure 3, the gas on the submerged hydrophobic surface grew within 300 s (Figure 3a–c), but then it stopped growing (from Figure 3d–h). The results indicate that evaporation at the liquid-gas interface was transient. Generally, the phenomenon ends when the partial pressure of water reaches the saturated vapor pressure.

**Figure 4:**
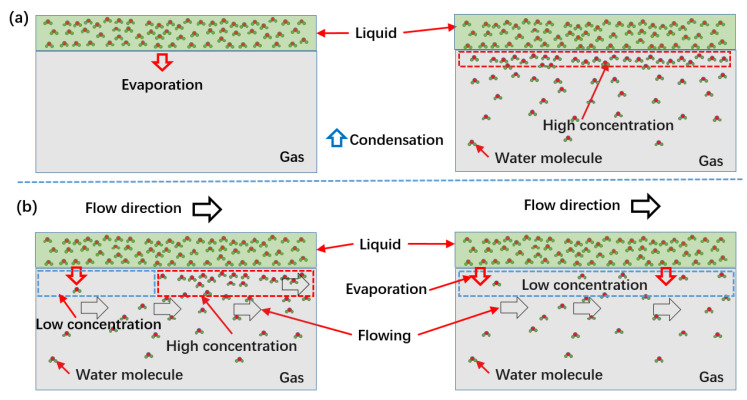
Schematic of evaporation at the liquid-gas interface, (**a**) for generation condition, the evaporation occurred when there was no water molecule in gas; the evaporation would stop due to the high concentration of water reaching to statured state; (**b**) under the action of flow, the water molecules in gas were washed away, the molecule with high concentration moved with flowing water; the low concentration could be sustained; therefore, the evaporation could keep happening.

As the results in Equation (5) and Figure 3 show, the evaporation rate declined gradually with increasing water concentration in the entrapped gas. As shown in Figure 4a, when the concentration of water in the trapped gas reached the saturated state, it achieved an equilibrium between evaporation and condensation at the liquid-gas interface. At equilibrium, the evaporation effect cannot be used to provide extra gas to sustain the nonwetted state of a submerged hydrophobic surface. However, according to Equation (5), if the partial pressure of water (Pv) in the entrapped gas stays lower than saturated vapor pressure (Ps), the equation rate is greater than zero, which indicates that the evaporation could go on. As shown in Figure 4b, once the gas layer reached high concentrations, gas was carried away by water flow, and the entrapped gas with a low concentration of water was achieved. Therefore, the partial pressure of water (Pv) in the trapped gas could remain low for a long time. Thus, under the action of flowing water, the concentration of water in entrapped gas cannot reach the saturated state, resulting in continuous evaporation.

**Figure 5:**
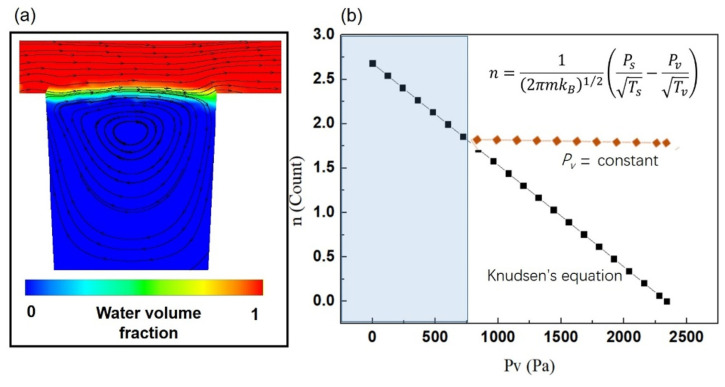
Influence of flowing water on the evaporation rate at the liquid-gas interface; (**a**) the flow field of hydrophobic structured surface; (**b**) the evaporation rate under the action of flowing water, when the concentration of water was kept at a low level, the partial pressure of water in entrapped gas could be considered a constant, and the evaporation rate didn’t descend with time.

To verify the influence of flowing water on the liquid-gas interface, a numerical simulation was carried out to investigate the flow field. As shown in Figure 5a, when water flows over the hydrophobic structured surface, the entrapped gas is sustained in the gap. According to the velocity distribution, the results indicate that the gas layer near the liquid-gas interface has velocity, which means that the gas layer with a high-water concentration was washed away by the flowing water. Therefore, the flowing water influenced the effects of the partial pressure of water on the evaporation rate. Because the field with a high concentration of water molecule was continually washed away by flowing water, the concentration of water was kept at a low level (Figure 5b). As a result, the partial pressure of water in entrapped gas could be considered a constant, and the evaporation rate didn’t descend with time.

**Figure 6:**
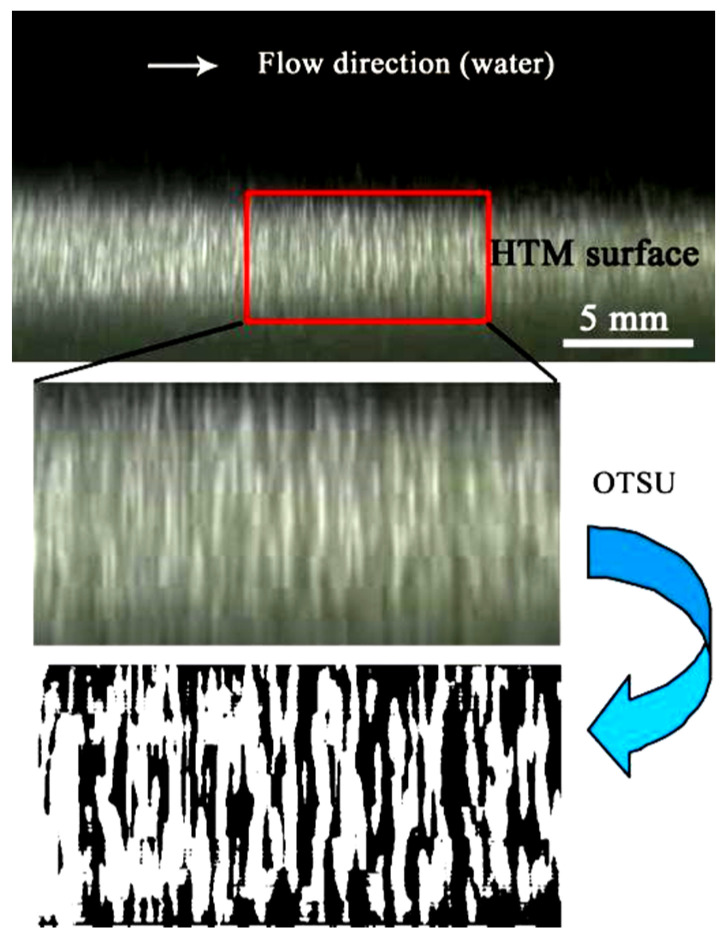
(color online) Image of gas on the HTM surface in flowing water, the selected part used for quantitative analysis of the growth rate and reduction rate of gas, and the image processed by the OTSU threshold method to differentiate the nonwetted area on the HTM surface.

Experiments have verified this proposed approach for gas replenishment by evaporation at the liquid-gas interface. As shown in Figure 6, when water perpendicularly flowed over the HTM surface, the result indicates that the phase structure corresponding to the bright area exists at the solid-liquid interface underwater [30,31,32], which confirms that hydrophobic grooves can hold gases within the microstructures in flowing water. Furthermore, the gas on the HTM surface was not static and instead fluctuated in the flowing water. The alternating variation of bright (caused by liquid-gas interface) and dark lines was observed (Figure 6), which indicates that the gas entrapped in HTMs was continuously carried away and then replenished by renewal gas without any energy or gas-providing device to supply it [32]. To measure this gas generation effect, as shown in Figure 6, a particular area (10 mm × 5 mm) was selected to quantifiably illustrate the gas coverage on the designed HTM surface, and then these chosen images were processed by a brightness thresholding algorithm to identify wetted (corresponding to the dark area) and nonwetted (the light area caused by gas) regions. To minimize the error caused by the definition of the threshold value, the OTSU algorithm (which depends on the image itself) was used to calculate this gray value. When the gray value of a pixel exceeded this threshold (141), calculated by the OTSU algorithm [33], the pixel was identified as nonwetted; otherwise, the pixel was identified as wetted.

**Figure 7:**
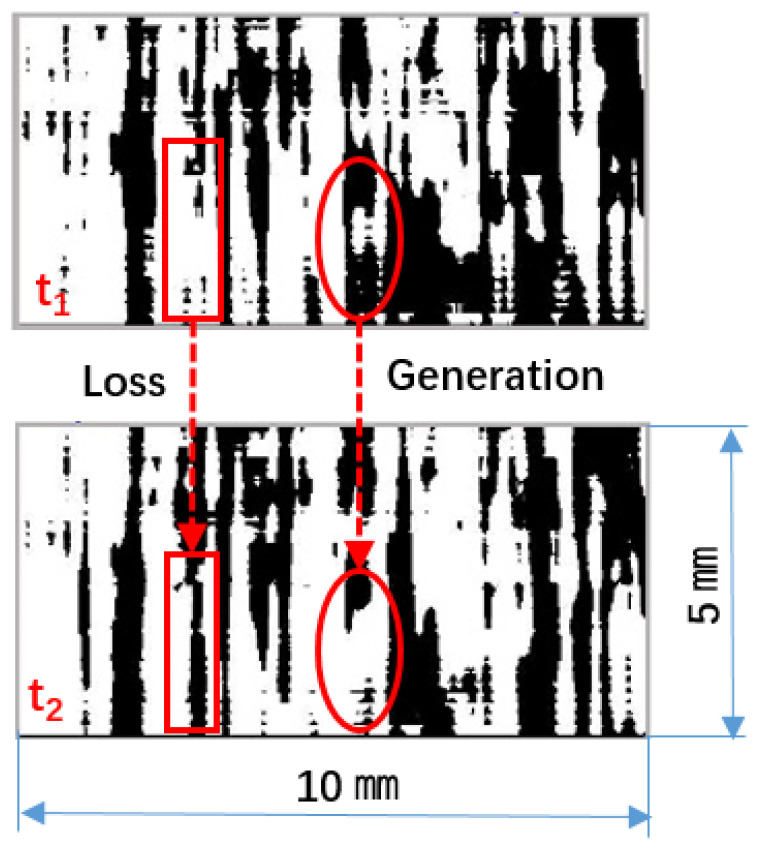
(color online) The gas coverage achieved by the threshold method on HTM surface in flowing water at different times, t_1_ and t_2_ is the different time point, the time interval between t_1_ and t_2_ is 0.125 s.

As shown in Figure 7, the results indicated that the gas coverage on the HTM surface was averagely stable, with immersion time in flowing water. In Figure 7, the fluctuation of the gas coverage with time showed that the gas was continually carried off (corresponding to the areas changing from light to dark) and was replenished by the renewal gas (corresponding to the regions changing from dark to light) in flowing water. The fluctuation of gas coverage was induced by the successive alternation of gas generation and gas loss [32].

The entrapped gas within the surface was essential for gas generation, and the flow velocity was also a key factor for gas generation. In the experiments, the fluctuation of gas coverage was induced by the successive alternation of gas generation and gas loss. Therefore, the gas automatic generation rate can be gauged by the sum of the new light pixels corresponding to the light area transformed from the dark during a unit time and it can be described as flow:

(8) $$G\%=\frac{Q_{1}+Q_{2}+Q_{3}+\ldots \ldots +Q_{n}}{ntQ_{total}}\times 100\%,$$

where *G* is the gas growth rate; *n* is the count of pairs of images; *t* is the interval time between two close images; *Q_i_* is the count of pixels becoming light from the *i*-th image to (*i* + 1)-th one; *Q_total_* is the total count of pixels in the selected image. Therefore, the reduction rate of gas (*R*) can be described as flow:

(9) $$R\%=\frac{q_{1}+q_{2}+q_{3}+\ldots \ldots +q_{n}}{ntQ_{total}}\times 100\%,$$

where, *q_i_* the count of pixels becoming dark from the *i*-th image to (*i* + 1)-th one.

**Figure 8:**
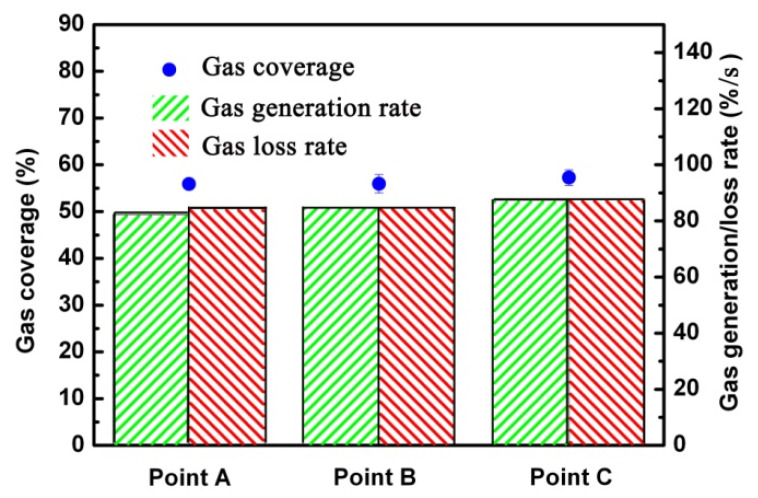
(color online) The gas coverage, gas generation rate, and gas loss rate at different sample positions with HTMs in flowing water at 5 m/s.

In the statistics, the time interval between each pair of images was set as 0.125 s, which is short enough to capture the progressive increase in a light area; 16 pairs of images were selected, which is enough to describe the fluctuation of the gas coverage. As shown in Figure 8, the gas generation rate is approximately equal to the gas loss rate, indicating that the loss of gas in flow was supplemented by the generation of gas. The gas coverage at three positions (positions A, B and C shown in Figure 2) was (55.89 ± 1.42)%, (55.95 ± 1.98)%, and (57.28 ± 1.65)%, respectively.

**Figure 9:**
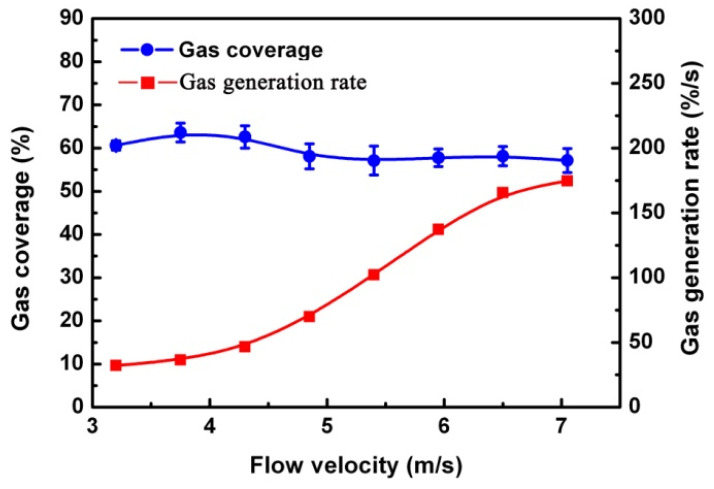
(color online) The gas coverage and generation rate on the HTM surface at different flow velocities.

The entrapped gas within the surface was essential for gas generation, and the flow velocity was also a key factor for gas generation. Although the higher flow velocities resulted in faster removal rates of gas [13] on the HTM surface, the gas-generation rate was enhanced with the flow velocity increase. This observation indicates that the accelerating generation of gas supplemented the increasing loss of gas with the increase in flow velocity, as shown in Figure 9, which finally results in a durable gas film on the HTM surface.

When there was no gas-providing device or energy supply, the evaporation at the liquid-gas interfaces above the microgrooves and the evolution of dissolved gas might cause the gas generation underwater. However, considering the evolution of dissolved gas, from the previous investigation, the time required for a gas bubble to grow ten times in size was several hundred seconds, even when the ratio of the dissolved gas concentration to the dissolved gas concentration for a statured solution was 1.25 [22]. Therefore, the growth rate of gas by evolution was too slow to achieve this phenomenon in our experiment. Therefore, gas generation in flowing water should primarily be attributed to the evaporation at the liquid-gas interface. To clarify this point further, the observations of gas on the HTM in the water with different gas contents were performed.

Here, ambient air was chosen as the solute gas, the gas concentration of a volume of water was accurately controlled by adequately exposing (72 h) the volume to a constant gas pressure for equilibration before the experiment. The steady-state gas concentration of liquids was described in terms of ambient pressure conditions through Henry’s Law [34]:

(10) $$p=k_{H}c$$

where *p* is the partial pressure of gas component above the liquid, *k_H_* is the Henry’s constant (material property) for a particular liquid and gas, and c is the steady-state concentration of gas within the liquid. The states of HTM surface under different gas concentrations (60 kPa, 101 kPa, 200 kPa) were investigated. The gas concentration of 60 kPa, 101 kPa, and 200 kPa corresponded to undersaturation, saturation, and supersaturation, respectively. According to the optical test results, the statistical results were shown in Table 2.

From the statistical results, both the gas coverage and the gas generation rate were not directly related to the air content in water, which conflicts with the precipitation of dissolved gas. Additionally, the entrapped gas (liquid-gas interface) was essential for regenerating gas, and the flow velocity significantly influenced the gas generation. Although the mechanism of gas generation needs to be fully characterized in future investigations, based on the results in this work, we surmise that the phenomenon of gas generation without any gas or energy supply should be attributed to the evaporation induced by flow at the liquid-gas interface.

**Figure 10:**
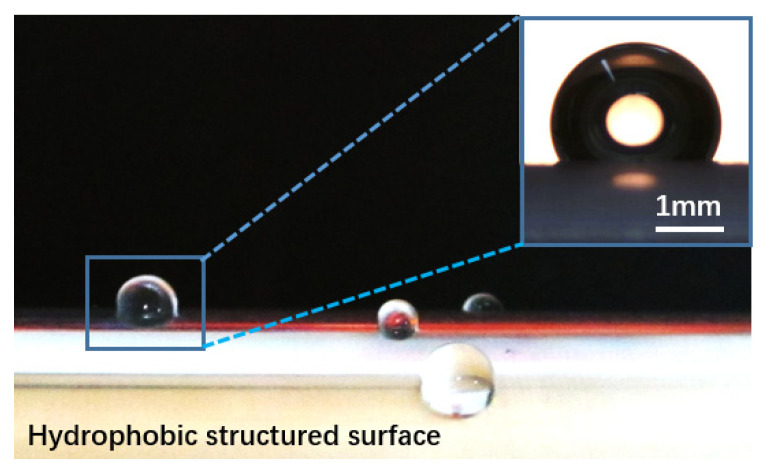
(color online) Images of a water droplet (5 μL) on the surfaces after the water tunnel experiments.

In previous investigation [18], when the surface was just taken out from the water, the surface was covered by discrete water layers which were sticky to the surface. It suggested that the entrapped air was mostly removed. After the flow test, our experiment immediately removed the HTM surface from the water, and surface-wetting conditions were examined again. The result in Figure 10 indicates that the sticky water residues on the HTM surface were much less, and, after the test, the surface was still hydrophobic. The HTM surface still sustained a noticeable amount of gas within the surface, even in fully turbulent flow for effective drag reduction under water. Although the entrapped gas might not be durable enough for several days or longer due to the dissolution [34], this HTM surface improved the longevity of trapped gas in turbulent flow due to the evaporation at the liquid-gas interface.

## 4. Conclusions

In summary, hydrophobic transverse microgrooves have been designed and fabricated to sustain air pockets in the gaps. The designed structures presented here successfully entrapped gas. Renewable gases were experimentally observed to form lubricating films at solid-liquid interfaces in flowing water. The mechanism of the gas generation should be attributed to evaporation at the liquid-gas interface induced by the flow. The results indicated that water molecules at the liquid-gas interface should escape to entrapped gas. When water content did not reach saturation levels, this phenomenon could be considered evaporation at the liquid-gas interface. With increasing water content in trapped gas, the evaporation rate at the liquid-gas interface descended gradually. Under the action of flowing, the substances containing high concentrations of water molecules at the liquid-gas interface were washed away, creating the low concentration of water molecules at the liquid-gas interfaces while evaporation continued to replenish the lost gas. Although further experimental and theoretical studies are needed to fully characterize the generation of gas and understand its underlying mechanism, this study can lead us down a new path toward achieving an effective underwater drag reduction approach and may further guide the development of related speed-enhancing and energy-saving technologies for underwater vehicles.

## Figures and Tables

**Table 1:** Results of hydrophobic treatment.

Surface Properties	Images	Contact Angle
smooth surface	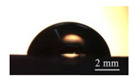	65°
smooth surface with FAS film	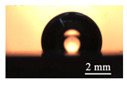	115 ± 2°
microgrooved surface with FAS-17 film	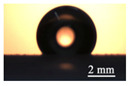	130°

**Table 2:** The effects of air content on the gas state on the HTM surface.

No.	Air Content (kPa)	State on HTM	Gas Coverage (%)	Gas Generation Rate (%)
1	60 (Undersaturation)	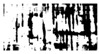	60.68 ± 3.39	92.48 ± 3.04
2	101 (Saturation)	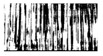	57.12 ± 3.33	102.48 ± 6.39
3	200 (Supersaturation)	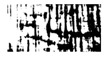	63.44 ± 4.75	95.78 ± 1.90

## Data Availability

Not applicable.
